# Patient Expression of Emotions and Neurologist Responses in First Multiple Sclerosis Consultations

**DOI:** 10.1371/journal.pone.0127734

**Published:** 2015-06-01

**Authors:** Lidia Del Piccolo, Erika Pietrolongo, Davide Radice, Carla Tortorella, Paolo Confalonieri, Maura Pugliatti, Alessandra Lugaresi, Andrea Giordano, Christoph Heesen, Alessandra Solari, AutoMS Project

**Affiliations:** 1 Department of Medicine and Public Health, University of Verona, Verona, Italy; 2 Department of Neuroscience, Imaging and Clinical Sciences, G. d’Annunzio University of Chieti-Pescara, Chieti, Italy; 3 Division of Epidemiology and Biostatistics, European Institute of Oncology, Milan, Italy; 4 Departments of Basic Medical Sciences, Neurosciences and Sense Organs, University of Bari, Bari, Italy; 5 Unit of Neuroimmunology, Foundation IRCCS Neurological Institute C. Besta, Milan, Italy; 6 Department of Clinical and Experimental Medicine, University of Sassari, Sassari, Italy; 7 Unit of Neuroepidemiology, Foundation IRCCS Neurological Institute C. Besta, Milan, Italy; 8 Institute for Neuroimmunology and Clinical MS Research, University Medical Center Hamburg-Eppendorf, Hamburg, Germany; Medical University of Innsbruck, AUSTRIA

## Abstract

**Background:**

Anxiety and depression are common in people with multiple sclerosis (MS), but data on emotional communication during MS consultations are lacking. We assessed patient expressions of emotion and neurologist responses during first-ever MS consultations using the Verona Coding Definitions of Emotional Sequences (VR-CoDES).

**Methods:**

We applied VR-CoDES to recordings/transcripts of 88 outpatient consultations (10 neurologists, four MS Italian centers). Before consultation, patients completed the Hospital Anxiety and Depression Scale (HADS). Multilevel sequential analysis was performed on the number of cues/concerns expressed by patients, and the proportion of reduce space responses by neurologists.

**Results:**

Patients expressed 492 cues and 45 concerns (median 4 cues and 1 concern per consultation). The commonest cues were verbal hints of hidden worries (cue type b, 41%) and references to stressful life events (type d, 26%). Variables independently associated with number of cues/concerns were: anxiety (HADS-Anxiety score >8) (incidence risk ratio, IRR 1.08, 95% CI 1.06-1.09; p<0.001); patient age (IRR 0.98, 95% CI 0.98-0.99; p<0.001); neurologist age (IRR 0.94, 95% CI 0.92-0.96; p=0.03); and second opinion consultation (IRR 0.72, 95% CI 0.60-0.86; p=0.007). Neurologists reacted to patient emotions by reducing space (changing subject, taking no notice, giving medical advice) for 58% of cues and 76% of concerns. Anxiety was the only variable significantly associated with ‘reduce space’ responses (odds ratio 2.17, 95% CI 1.32-3.57; p=0.003).

**Conclusions:**

Patient emotional expressions varied widely, but VR-CoDES cues b and d were expressed most often. Patient anxiety was directly associated with emotional expressions; older age of patients and neurologists, and second opinion consultations were inversely associated with patient emotional expression. In over 50% of instances, neurologists responded to these expressions by reducing space, more so in anxious patients. These findings suggest that neurologists need to improve their skills in dealing with patient emotions.

## Introduction

Multiple sclerosis (MS) is a chronic, degenerative disease of the central nervous system that affects women 2–3 times more often than men, and is the leading cause of non-traumatic neurologic disability in young adults [1]. Variable manifestations and unpredictable prognosis characterize the condition, which impacts on sufferers’ mental health [2], quality of life [3], and coping with the disease [4]. Depending on the ascertainment method and study population, the prevalence of depression in MS is 11–50% [5–10]—higher than in other chronic conditions including neurological disorders [11]. Anxiety has been less extensively assessed in MS than depression; its prevalence varies from 20 to 40% [7–10]. Depression and anxiety symptoms are often associated in people with MS [8,10]. Notwithstanding this, neurologists are usually less concerned with the emotional aspects of the disease than its ‘physical’ manifestations [12].

The effectiveness of patient-physician communication improves when physicians recognize and respond empathically to patient concerns [13,14]. Ability to recognize and manage patients’ emotional needs is a key feature of patient-centered care and is associated with a variety of positive patient outcomes [15], including adherence to long-term treatment [16–18]. Detecting and responding to negative emotions is also important for diagnostic and therapeutic purposes [15]. Nonetheless, studies on emotional communication in various settings indicate that physicians often miss patient worries and concerns, and respond to them by providing biomedical information, or nonspecific arguments [19]. To our knowledge only one study [4] on recurrent expressions of emotions in MS patients, and how they were dealt with by the healthcare professional, has been published.

In the present study we aimed to appraise patient expressions of emotion during outpatient consultations and neurologist responses to those expressions, using the Verona Coding Definitions of Emotional Sequences (VR-CoDES) instrument [20,21]. Patient and neurologist characteristics likely to affect both outcomes were also explored.

This study is part of the international AutoMS project (Autonomy preferences, risk knowledge and decision making performance in MS patients; www.automsproject.org).

## Methods

### Participants and procedures

Study procedures and participant characteristics are described elsewhere [22]. Briefly, we recorded consultations with a neurologist occurring at four Italian MS centers. Eligible patients were adults able to give informed consent and who were attending for first-ever consultation (patients already being followed were excluded). Before the consultation, patients completed the Hospital Anxiety and Depression Scale (HADS) [23,24]. The consultations were unobtrusively audio-taped and transcribed verbatim.

Transcripts and audio recordings were coded according to VR-CoDES [20,21]. After EP had received two day’s training with LDP (one of the VR-CoDES authors), consultations were initially coded independently by EP and LDP, with differences resolved by discussion. After 12 consultations had been coded, only minor coding discrepancies occurred and coding was completed by EP, with only exceptional involvement by LDP.

### Ethics statement

All study patients and neurologists gave written consent to participate, and for the consultation to be recorded. The protocol was approved by the Ethics Committees of the following hospitals: Foundation IRCCS Neurological Institute C. Besta, Milan; G. d’Annunzio University of Chieti-Pescara, Chieti; University of Bari; University of Sassari; all in Italy.

### Measures

#### Verona Coding Definitions of Emotional Sequences (VR-CoDES)

The VR-CoDES [21] categorizes patient expressions of emotion as ‘cues’ (verbal or nonverbal hints, which suggest an underlying unpleasant emotion) and ‘concerns’ (clear, unambiguous verbalizations of unpleasant current or recent emotions with or without indicating their importance). Cues are further divided into seven sub-categories (Table 1) [20].

**Table 1:** The seven sub-categories of cues in the Verona Coding Definitions of Emotional Sequences (VR-CoDES) [21].

a. Words or phrases in which the patient uses vague or unspecified words to describe his/her emotions.
b. Verbal hints to hidden concerns (emphasizing, unusual words, unusual description of symptoms, profanities, exclamations, metaphors, ambiguous words, double negatives, expressions of uncertainties and hope).
c. Words or phrases which emphasize (verbally or non-verbally) physiological or cognitive correlates (regarding sleep, appetite, physical energy, excitement or motor slowing down, sexual desire, concentration) of unpleasant emotional states. Physiological correlates may be described by words such as weak, dizzy, tense, restless, or by reports of crying whereas cognitive correlates may be described by words such as poor concentration or poor memory.
d. Neutral expressions that mention issues of potential emotional importance which stand out from the narrative background and refer to stressful life events and conditions. This applies to non-verbal emphasis of the sentence, abrupt introduction of new content, pauses before or after the expression, or to a patient-elicited repetition of a previous neutral expression in subsequent turns.
e. A repetition, with very similar words, of an expression said in a previous turn by the patient.
f. Non-verbal clear expressions of negative or unpleasant emotions (crying), or hints to hidden emotions (sighing, silence after provider question, trembling voice, frowning, etc.).
g. A clear and unambiguous expression of a concern, e.g., a previous mental state, a previous worry or fear, referring to a past episode, of more than four weeks ago or without a clear time frame.

Cues/concerns can be expressed spontaneously by the patient or elicited by the health provider. Those expressed spontaneously aim to bring up topics that the health provider has so far neglected, or not adequately explored. Health provider responses to cues/concerns are classified according to whether they provide space or reduce space for further exploration of the cue/concern [21,25,26]. The VR-CoDES manuals are available at: http://www.each.eu/verona-coding-systems.

#### Hospital Anxiety and Depression Scale (HADS)

HADS is a self-assessed questionnaire consisting of 14 multiple choice (0–3 Likert scale) items probing symptoms of anxiety (7 items) and depression (7 items). Anxiety (HADS-A) and depression (HADS-D) scores range from 0 (no symptoms) to 21 (most severe symptoms) [23]. The instrument has robust psychometric properties, and has been validated in several languages, including Italian [24]. Furthermore, by omitting items assessing somatic symptoms, and thereby limiting false positive findings, the scale is suitable for use in persons with medical conditions [27]. HADS is widely-used in MS patients, and cutoff scores of 8 or above are considered an accurate indicator of major depression (HADS-D) or generalized anxiety disorder (HADS-A) in this population [28].

The data which forms the basis for the analysis can be found in S1 Dataset.

### Statistical analysis

Patient and neurologist characteristics were summarized by counts and percentages (categorical variables) or means and standard deviations (SDs), or medians with interquartile ranges (IQRs) (continuous variables). Group comparisons employed the chi-square test, Fisher’s exact test (categorical variables) or the Wilcoxon rank-sum test (continuous variables).

We assessed variables associated with number of expressed cues/concerns using hierarchical Poisson regression, with results presented as incidence rate ratios (IRRs) with 95% confidence intervals (CIs). Independent variables comprised: patient age, gender, education (primary vs. secondary/college), HADS-A, HADS-D, and diagnosis (MS or clinically isolated syndrome [CIS] vs. other conditions); neurologist age, gender, and years of MS experience; and whether second opinion consultation. We also assessed variables associated with neurologists’ immediate verbal responses to emotions (outcome variable: ‘reduce space’ response, same independent variables as above) using hierarchical logistic regression with results presented as odds ratios (ORs) with 95% CIs. In both types of hierarchical regression analysis, neurologists’ characteristics were nested within the MS center, and consultation length was entered as exposure (offset) variable [29,30].

The analyses were done with SAS 9.3 (SAS Institute Inc., Cary, NC, USA). All statistical tests were two-tailed; differences were considered significant at an alpha level of 0.05.

## Results

### Participants

Of the 117 patients approached, 25 (21%) refused and four audio recordings were incomplete and could not be rated; thus 88 consultations were transcribed and analyzed. Mean patient age was 37.5 years; 58 (66%) were women, and 63 (72%) had MS or CIS. The remaining participants had suspected MS, radiologically isolated syndrome, optic neuritis or other diagnoses (Table 2). Twenty five percent of patients came for a second opinion, 67% were employed, and 19% had a high level of education. Thirty-eight percent of patients had HADS-A score above threshold, 17% had HADS-D score above threshold, and 14% had both anxiety and depression scores above threshold.

**Table 2:** Characteristics of the 88 patients participating in the study.

Characteristic	Sub-characteristic	
Women (%)		58 (66)
Mean age (years), SD (min-max)		37.5, 11.4 (20–69)
Diagnosis (%)	MS/CIS	63 (72)
	Other condition^a^	25 (28)
Index problem: second opinion (%)		22 (25)
Highest level of education (years) (%)	Primary	23 (26)
	Secondary	48 (55)
	College/University	17 (19)
Current employment status (%)	Employed, full-time	46 (53)
	Employed, part-time	12 (14)
	Home employment	11 (13)
	Student	8 (9)
	Unemployed	6 (7)
	Retired (age)	3 (3)
	Disability pension	1 (1)
Disease type (n = 63 MS/CIS patients) (%)	First episode/relapsing-remitting	56 (89)
	Relapsing-progressive/chronic progressive	7 (12)
Median EDSS score (IQR) (n = 63 MS/CIS patients)		2.0 (1.5–3.5)
Mean HADS, SD (min-max) (n = 87 patients with valid scores)	Anxiety (HADS-A)	7.8, 4.0 (0–19)
	Depression (HADS-D)	4.5, 3.5 (0–14)

^a^Encephalopathy/myelopathy (n = 10); suspected MS (n = 9); radiologically isolated syndrome (n = 2); optic neuritis (n = 1); headache (n = 1); chronic inflammatory demyelinating polyneuropathy (n = 1); facial spasm (n = 1). The diagnosis was provided by the neurologist on the case report form.

MS is multiple sclerosis, CIS is clinically isolated syndrome, EDSS is Expanded Disability Status Scale, HADS is Hospital Anxiety and Depression Scale.

Median neurologist age was 47.5 years (range 30–51), 50% were women, and median experience with MS was 7.5 years (range 3–24). More details on participating centers, consultation type, and neurologist characteristics are reported elsewhere [22].

### Patient emotional expressions and immediate neurologist responses

Overall we detected 492 VR-CoDES cues and 45 concerns, with a median of 4 cues (IQR 1–5) and 1 concern (IQR 1–2) per consultation. As shown in Table 3, verbal hints to hidden concerns (b type cues) were the most common cues (41%), followed by neutral expressions referring to stressful life events (d cues) (26%). Cue types a, c, e and f constituted less than 10% of all cues. Most cues and concerns were expressed spontaneously by patients, the only exception being c cues (related to physiological correlates of emotion). Neurologists reacted to patient emotions (58% of cues, and 76% of concerns) mainly by reducing space—i.e. by providing medical advice, switching topic, or ignoring the content of the cue; this happened significantly more often after the patient expressed a concern, and also after the patient referred to stressful life events/situations (d cue) (Table 3).

**Table 3:** Distribution of cues/concerns according to whether spontaneous or neurologist elicited and according to neurologist immediate response (reduce vs. provide space).

		Origin			Neurologist’s response		
		No (row %)			No (row %)		
	No cues/concerns (column %)	Spontaneous	Neurologist-elicited	P value*	Reduce space	Provide space	P value**
Concern	45 (8)	34 (76)	11 (24)	<0.001	34 (76)	11 (24)	<0.001
Cue a	36 (7)	23 (64)	13 (36)	0.10	18 (50)	18 (50)	1.0
Cue b	204 (38)	138 (68)	66 (32)	<0.001	105 (51)	99 (49)	0.7
Cue c	31 (6)	14 (45)	17 (55)	0.59	15 (48)	16 (52)	1.0
Cue d	129 (24)	106 (82)	23 (18)	<0.001	90 (70)	39 (30)	<0.001
Cue e	6 (1)	4 (67)	2 (33)	0.41	2 (33)	4 (67)	0.7
Cue f	36 (7)	26 (72)	10 (28)	0.008	24 (67)	12 (33)	0.06
Cue g	50 (9)	39 (78)	11 (22)	<0.001	30 (60)	20 (40)	0.2
Totals	537	384 (71)	153 (29)	<0.001	318 (59)	219 (41)	<0.001

* Spontaneous vs. neurologist-elicited expression

** Reduce vs. provide space response

### Characteristics associated with patient emotional expressions and neurologist responses

Patient variables significantly associated with the total number of cues/concerns in univariate analyses (Table 4) were age (negative association, IRR 0.99, 95% CI 0.98–1.00), above-threshold anxiety (IRR 1.05, 95% CI 1.03–1.06), and above-threshold depression (IRR 1.02, 95% CI 1.00–1.04). Neurologist variables associated with total number of cues/concerns expressed were age (negative association, IRR 0.95, 95% CI 0.93–0.98), being a woman (IRR 1.65, 95% CI 1.45–1.88), and years of MS experience (IRR 1.04, 95% CI 1.03–1.05). Second opinion consultations were also negatively associated with number of expressed cues/concerns (IRR 0.56, 95% CI 0.46–0.67). In the multivariate hierarchical Poisson model (Table 4), the variables that remained significant were patient age (IRR 0.98, 95% CI 0.98–0.99), above-threshold anxiety (IRR 1.08, 95% CI 1.06–1.09), neurologist age (IRR 0.94, 95% CI 0.92–0.96), and second opinion consultation (IRR 0.72, 95% CI 0.60–0.86).

**Table 4:** Characteristics associated with patient cues and concerns in univariate and multivariate hierarchical Poisson models.

		Univariate			Multivariate		
Patient characteristics		IRR	95% CI	P-Value	IRR	95% CI	P-Value
Age (years)		**0.99**	**0.98–1.00**	**0.002**	**0.98**	**0.98–0.99**	**<0.001**
Education	Primary	Reference					
	Secondary/College+	1.92	1.14–3.26	0.07			
Gender	Men	Reference					
	Women	1.18	1.00–1.37	0.10			
HADS-A		**1.05**	**1.03–1.06**	**<0.001**	**1.08**	**1.06–1.09**	**<0.001**
HADS-D		**1.02**	**1.00–1.04**	**0.041**	0.99	0.97–1.00	0.17
Diagnosis	Other conditions	Reference					
	MS/CIS	0.93	0.81–1.08	0.40			
**Neurologist characteristics**							
Age (years)		**0.95**	**0.93–0.98**	**0.02**	**0.94**	**0.92–0.96**	**0.03**
MS experience (years)		**1.04**	**1.03–1.05**	**0.002**	0.99	0.97–1.01	0.30
Gender	Men	Reference			Reference		
	Women	**1.65**	**1.45–1.88**	**0.002**	1.20	1.02–1.42	0.16
Response	Provide space	Reference					
	Reduce space	1.08	0.94–1.24	0.31			
**Other characteristics**							
Second opinion consultation	No	Reference			Reference		
	Yes	**0.56**	**0.46–0.67**	**0.002**	**0.72**	**0.60–0.86**	**0.007**
Consultation length (minutes)		*Exposure*					

IRR is incidence rate ratio, and 95% CI the IRR confidence interval; MS is multiple sclerosis; HADS-A is Hospital Anxiety and Depression Scale Anxiety score; HADS-D is HADS Depression score.

Above-threshold anxiety was the only variable significantly associated with a ‘reduce space’ response by the neurologist (OR 2.17, 95% CI 1.32–3.57; S1 Table). No multivariable model was run.

## Discussion

We investigated how MS patients manifest their emotions (as cues or explicitly expressed concerns) during first-ever consultations at MS centers, and how neurologists reacted to those expressions. Variables associated with patient emotions and neurologist responses were also explored. We found that patients expressed a median of four cues and one concern during the consultation. This is consistent with the three-to-four cues/concerns expressed by cancer patients [31–33]; however patients with fibromyalgia [34] or psychiatric conditions [35] expressed more cues/concerns.

The most frequent cues identified (Table 1) were b cues (41%) followed by d cues (26%), all others were 10% or below. B cues are common in all studies that use VR-CoDES [31,34,35] perhaps because using colorful or figurative language (Table 1) is the easiest and most immediate way of expressing an affective state without mentioning emotions (Cit. 1 “*There was a period… perhaps for about a month when I felt pain… back pain…*. *and I felt tired*. *It was all getting too much*. *I thought it was because I was working too many hours and needed to rest*. *Needed lots of rest*. *But then I found out [sigh] it wasn’t rest I needed*.”

Cit. 2 “*It felt like my head was going to explode*. *My brain would be floating… like my head was full of water and my brain was there floating in it*.*”)*

Our study is unusual in finding a high percentage of d cues. Zhou et al. [31] found that d cues were the second most common cues (at 16%) in head and neck cancer survivors, but not as common as in our study.

This could reflect the high impact of MS on everyday life (Cit. 3 Doctor: “*Why have you decided to be followed at this center [instead of Milan]*?*”* Patient: *“Well*, *I’m still thinking about that*, *but it’s getting too stressful to go to Milan every three months*. *It doesn’t fit with my studies*, *my personal life… And I also get very tired… every day I feel exhausted*.” Cit. 4: “*This year I’ve felt really distressed*, *but I’ve tried to keep active by going to the gym anyway*. *But is it OK to do sports*?”).

We found that HADS-A score above threshold was strongly and independently associated with the number of cues and concerns expressed by our patients. Other studies have also found that emotionally distressed patients expressed more cues/concerns [36–39].

Regrettably, 75% of our patient expressions of concern were met by reduction of space, and this was particularly the case for anxious patients. Neurologists often switched topic, devalued emotions (Cit. 5: Patient: *“I’m so lacking in energy*. *I can’t get up*, *I feel so low…”* Doctor: *“That’s strange*. *Cortisone usually picks you up*. *You shouldn’t be feeling so low”*), ignored the emotion (Cit. 6: Patient: *“Yeah*, *cortisone makes me feel so nervous*, *I’m always anxious when on cortisone…”* Doctor: *“Perhaps I should give you a 1*.*5 g cortisone boost for three days*, *and see how you go…”*) or gave generic reassurance (Cit. 7: Patient: *“I often wonder why this had to happen to me*. *It’s horrible*.*”* Doctor: *“Yes*, *but*, *it’s not the end of the world*. *It may seem serious*, *but I have many MS patients who lead normal lives…”*). Similar findings were also reported by Pollak et al. [40] in the cancer setting: in particular they found that when patients expressed negative emotions, physicians responded 73% of times with a closure statement effectively discouraging further disclosure. Similarly, in the hospital setting across different medical specialties Mjaaland et al. [41] found that most physician responses to patient cues/concerns did not include follow up or exploration. As in our study, concerns more often than cues were not given space. The authors noted [41] that while concentrating on medical aspects, hospital doctors might not realize that emotional exploration was sometimes necessary to understand the disease in a patient and tailor management to that patient’s needs.

It is noteworthy that consultations with younger patients and younger neurologists were associated with greater expression of emotions, as also reported by Butow et al. [42] in the cancer setting, and Del Piccolo et al. [35] in the psychiatric setting.

Finally, second-opinion consultations were associated with fewer expressions of emotion. This may be because the patient is more concerned with ‘technical’ issues like other possible diagnoses and treatment, so less emotional content might be expected.

Regarding the origin of emotional expression (spontaneous or neurologist-elicited), 75% of cues were spontaneous, indicating that emotional topics were rarely raised by neurologists, further indicating that emotional aspects were not for them a pressing concern. As noted above, this may be because they were concentrating on medical aspects in a consultation of limited duration. Giving information and advice (which in VR-CoDES is coded as reduction of space) is at the heart of the medical consultation; nevertheless helping patients to verbalize their feelings facilitates emotion regulation [43], predicts competent coping [44], generates greater patient satisfaction with interpersonal care [14], and increases collaboration [36].

Limitations of the present study are that a restricted number of consultations (total 88) was recorded and only 10 neurologists were involved. Furthermore the consultations were audio recorded, so some non-verbal expressions will have been missed. We did not assess whether neurologist skill in dealing with emotion affected patient satisfaction with the consultation. After the consultation, patients completed the Patient Involvement in Care Scale (PICS) [45], as a measure of patient-assessed involvement in decision-making (and thus their satisfaction with this aspect) [22]. However, our findings and those of a study in the psychiatry setting indicate that PICS is not reliable [22,46]. We therefore decided not to include PICS results in the present study. In our opinion an adequate patient-reported measure of patient satisfaction with consultations is not yet available [14].

A strength of the study is that all consultations were first contact between the patient and the neurologist (thus follow-up consultations for treatment review or other reason were excluded). To our knowledge, no studies on emotional communication during neurological consultations have been published previously.

To conclude, our findings complement those of our previous study on shared decision-making in the same consultations [22] which suggested the need to empower Italian MS neurologists with better communication and shared decision-making skills. In particular our findings indicate that physicians too often fail to respond sensitively to the emotional cues expressed by their patients, while other data suggest that sensitive responses are essential for effective shared decision making [47]. An integrated approach that retains all the elements of evidence-based medicine but adds on shared decision-making is essential for quality health care, and should be taught at all levels of medical training [48].

## Supporting Information

S1 Dataset Raw data from patients’ case record form, Hospital Anxiety and Depression Scale (HADS), and Verona Coding Definitions of Emotional Sequences (VR-CoDES). (XLS)

S1 Table Characteristics associated with neurologist ‘reduce space’ response in univariate logistic regression. (DOCX)
