# Supplementary material for: Patient Expression of Emotions and Neurologist Responses in First Multiple Sclerosis Consultations
Source: PLoS One. 2015 Jun 1;10(6):e0127734. doi: 10.1371/journal.pone.0127734 (PMC4452259; doi:10.1371/journal.pone.0127734)
Supplement: S1 Table — (DOCX) [file pone.0127734.s002.docx]

**Table S1.** **Characteristics associated with neurologist ‘reduce space’ responses in univariate hierarchical logistic regression.**

| **Patient characteristics** |  | **No (%)** | **OR** | **95% CI** | **P-Value** |
| --- | --- | --- | --- | --- | --- |
| Age (years) |  | – | 0.99 | 0.97-1.01 | 0.30 |
| Education | Primary | 217 (60) | Reference |  |  |
|  | Secondary/College+ | 101 (57) | 0.88 | 0.30-2.56 | 0.81 |
| Gender | Men | 71 (51) | Reference |  |  |
|  | Women | 247 (62) | 1.54 | 0.88-2.63 | 0.13 |
| HADS-A | ≤ 8 | 81 (46) | Reference |  |  |
|  | > 8 | 237 (65) | **2.17** | **1.31-3.57** | **0.003** |
| HADS-D | ≤ 8 | 49 (56) | Reference |  |  |
|  | > 8 | 269 (60) | 1.14 | 0.60-2.17 | 0.70 |
| Diagnosis | Other conditions | 160 (62) | Reference |  |  |
|  | MS/CIS | 158 (57) | 0.81 | 0.48-1.39 | 0.44 |
| **Neurologist characteristics** |  |  |  |  |  |
| Age (years) |  | – | 1.04 | 0.99-1.10 | 0.10 |
| MS experience (years) |  | – | 0.99 | 0.96-1.03 | 0.80 |
| Gender | Men | 107 (58) | Reference |  |  |
|  | Women | 211 (60) | 1.10 | 0.65-1.85 | 0.72 |
| **Other characteristics** |  |  |  |  |  |
| Second opinion consultation | No | 250 (61) | Reference |  |  |
|  | Yes | 68 (54) | 0.76 | 0.45-1.41 | 0.43 |
| Consultation length (minutes) |  | *–* | *Exposure* | – | – |

OR is odds ratio, and 95% CI the OR confidence interval; MS is multiple sclerosis; HADS-A is Hospital Anxiety and Depression Scale Anxiety score; HADS-D is HADS Depression score.
